# *SPT20* Regulates the Hog1-MAPK Pathway and Is Involved in *Candida albicans* Response to Hyperosmotic Stress

**DOI:** 10.3389/fmicb.2020.00213

**Published:** 2020-02-21

**Authors:** Lianfang Wang, Ruilan Chen, Qiuting Weng, Shaoming Lin, Huijun Wang, Li Li, Beth Burgwyn Fuchs, Xiaojiang Tan, Eleftherios Mylonakis

**Affiliations:** ^1^Department of Respiratory and Critical Care Medicine, Chronic Airways Diseases Laboratory, Huiqiao Medical Center, Nanfang Hospital, Southern Medical University, Guangzhou, China; ^2^The Second Clinical College of Guangzhou University of Chinese Medicine, Guangzhou, China; ^3^Department of Intensive Care Unit, Fangcun Branch of Guangdong Provincial Hospital of Chinese Medicine, Guangzhou, China; ^4^Guangdong Provincial Academy of Chinese Medical Sciences, Guangzhou, China; ^5^Department of Respiratory, Longhua District People’s Hospital, Shenzhen, China; ^6^Huiqiao Medical Center, Nanfang Hospital, Southern Medical University, Guangzhou, China; ^7^Department of Medicine, Division of Infectious Diseases, Rhode Island Hospital, Warren Alpert Medical School of Brown University, Providence, RI, United States

**Keywords:** *Candida albicans*, glycerol, Hog1-MAPK, osmotic stress, *SPT20*

## Abstract

*Candida albicans* is the most common fungal pathogen and relies on the Hog1-MAPK pathway to resist osmotic stress posed by the environment or during host invasions. Here, we investigated the role of *SPT20* in response to osmotic stress. Testing a *C. albicans spt20*Δ/Δ mutant, we found it was sensitive to osmotic stress. Using sequence alignment, we identified the conserved functional domains between CaSpt20 and ScSpt20. Reconstitution of the Spt20 function in a *spt20*Δ*/CaSPT20* complemented strain found *CaSPT20* can suppress the high sensitivity to hyperosmotic stressors, a cell wall stress agent, and antifungal drugs in the *Saccharomyces cerevisiae spt20*Δ/Δ mutant background. We measured the cellular glycerol accumulation and found it was significantly lower in the *C. albicans spt20*Δ/Δ mutant strain, compared to the wild type strain SC5314 (*P* < 0.001). This result was also supported by quantitative reverse transcription-PCR, which showed the expression levels of gene contributing to glycerol accumulation were reduced in *Caspt20*Δ/Δ compared to wild type (*GPD2* and *TGL1*, *P* < 0.001), while *ADH7* and *AGP2*, whose expression can lead to glycerol decrease, were induced when cells were exposed to high osmolarity (*ADH7*, *P* < 0.001; *AGP2*, *P* = 0.002). In addition, we tested the transcription levels of Hog1-dependent osmotic stress response genes, and found that they were significantly upregulated in wild type cells encountering hyperosmolarity, while the expression of *HGT10*, *SKO1*, *CAT1*, and *SLP3* were not induced when *SPT20* was deleted. Although the transcript of *ORF19.3661* and *ORF19.4370* in *Caspt20Δ/Δ* was induced in the presence of 1 M NaCl, the levels were less than what was observed in the wild type (*ORF19.3661*, *P* = 0.007; *ORF19.4370*, *P* = 0.011). Moreover, the deletion of *CaSPT20* in *C. albicans* reduced phosphorylation levels of Hog1. These findings suggested that *SPT20* is conserved between yeast and *C. albicans* and plays an important role in adapting to osmotic stress through regulating Hog1-MAPK pathway.

## Introduction

*Candida albicans* can be isolated from oral-pharyngeal, gastrointestinal, and urogenital tracts (Calderone and Fonzi, 2001), and has emerged as one of the most common causes of nosocomial bloodstream infections (Wisplinghoff et al., 2004). In order to cause colonization and infection, this successful opportunistic pathogen has to overcome environmental challenges, such as host immune defenses, nutrient limitation, competition with resident microbiota, and physiological extremes including: pH, osmotic, and oxidative stresses (Calderone and Fonzi, 2001; Marotta et al., 2013; Dong et al., 2015). *C. albicans* has developed a series of complex mechanisms to respond to these challenges.

The high osmolarity glycerol 1 mitogen activated protein kinase signaling transduction pathway, also known as the Hog1-MAPK pathway, can regulate responses to oxidative, osmotic, and heavy metal stress (Enjalbert et al., 2006). Therefore, the Hog1 signal transduction pathway is crucial for *C. albicans* cells during exposure to stressors encountered during pathogenesis (Alonso-Monge et al., 1999). When cells encounter hyperosmotic conditions, they rapidly trigger the Hog1-MAPK pathway to regulate Hog1-dependent osmotic stress response genes, and the synthesis and accumulation of glycerol. Glycerol is an important compatible cellular solute. When cells encounter osmotic challenge, they can make a comparable change in glycerol content to offset the increasing external osmolarity, thus buffering the osmotic change to maintain normal cell volume and enable survival (Reed et al., 1987).

This important pathway can be influenced by other genes. *SPT20*, an important component of the SAGA complex, helps to maintain the structural integrity of the SAGA complex (Grant et al., 1997; Sterner et al., 1999), controls about 10% of gene expression (Lee et al., 2000), and is highly conserved in eukaryote cells (Sellam et al., 2009). The interaction between Spt20 and Hog1 is essential for osmotic adaption (Zapater et al., 2007). More specifically, when cells were subjected to osmotic stress, Hog1 was activated and bound to osmostress promoters, then recruited SAGA complex components (including Spt20). Further experiment showed Hog1 co-precipitated the Spt20, suggesting that Hog1 associates with Spt20 (Zapater et al., 2007). The activation of human Hog1 did not correlate with an increased recruitment of hSpt20 subunit under endoplasmic reticulum stress (Nagy et al., 2009). However, p38IP, the human ortholog of the yeast Spt20, can directly bind to p38 and is required for the activation of the mammalian ortholog of Hog1 (Zohn et al., 2006). These previous studies indicate that further work is still needed to explore the interaction between Spt20 and Hog1.

In our previous research, we reported that *SPT20* was involved in toleration to high osmotic stress, revealing it was associated with *C. albicans* virulence (Tan et al., 2014). However, the association between *CaSPT20* and Hog1-MAPK signaling pathway in *C. albicans* is still poorly understood. In this study, we perform quantitative reverse transcription-PCR (qRT-PCR) and western blotting to interrogate the relationship between *SPT20* and Hog1-MAPK pathway in *C. albicans*. We describe the conserved role of *SPT20* between *S. cerevisiae* and *C. albicans* and report, for the first time, that *SPT20* takes part in the *C. albicans* response to hyperosmotic stress by regulating the Hog1-MAPK pathway.

## Materials and Methods

### Yeast Strains and Growth Conditions

Wild type *C. albicans* strain SC5314, the *spt20Δ/Δ* null mutant, the *spt20Δ/SPT20* reconstituted strain, and *S. cerevisiae* wild type BY4741 were grown in YPD medium (1% yeast extract, 2% peptone, 2% dextrose) at 30°C with shaking. The *Saccharomyces cerevisiae spt20Δ* mutant strain (LCT1) was cultured in YPD medium supplemented with G418 (Sigma-Aldrich, Shanghai, China). Ampicillin-resistant *E. coli* was cultured in LB medium with 100 μg/mL ampicillin at 37°C. Strains with *pYES-CaSPT20-V5* or *pYES2.1/V5-His-TOPO* plasmids were cultured in Sc-Ura3 media. All strains were cultured to logarithmic growth stage.

*C. albicans* and *S. cerevisiae* strains used in this study are listed in Table 1. All *C. albicans* strains were derived from the wild type strain SC5314. All *S. cerevisiae* strains were derived from the wild type BY4741.

**TABLE 1:** Strains used in this study.

**Microbial**	**Strains**	**Genotype**	**Reference or source**
*E.coli*	*DH-5*α	*F*-, *Δ80dlacZ*Δ*M15*, Δ(*lacZYA-argF*)*U169*, *deoR*, *recA1*, *endA1*, *hsdR17* (*rk-*, *mk*+), *phoA*, *supE44*, λ*-*, *thi-1*, *gyrA96*, *relA1*	From Takara
*S.cerevisiae*	BY4741	*MAT****a*** *his3Δl leu2Δ0 metl5Δ0 ura3Δ0*	From Merck
*S.cerevisiae*	LCT1	*MAT****a*** *his3Δl leu2Δ0 metl5Δ0 ura3Δ0:spt20:kanMX6*	This study
*S.cerevisiae*	LCT2	*MAT****a*** *his3Δl leu2Δ0 metl5Δ0 ura3Δ0:spt20:kanMX6 pYES2.1/V5-His-TOPO*	This study
*S.cerevisiae*	LCT3	*MAT****a*** *his3Δl leu2Δ0 metl5Δ0 ura3Δ0:spt20:kanMX6 pYES- CaSPT20- V5*	This study
*S.cerevisiae*	LCT4	*MAT****a*** *his3Δl leu2Δ0 metl5Δ0 ura3△0 pYES2.1/V5-His-TOPO*	This study
*C. albicans*	SC5314	Wild type	From Eleftherios Mylonakis
*C. albicans*	*spt20Δ/Δ*	*spt20Δ:FRT/spt20Δ:FRT*	From Eleftherios Mylonakis
*C. albicans*	*spt20Δ/SPT20*	*spt20Δ:FRT/SPT20-FRT*	From Eleftherios Mylonakis
*C. albicans*	*hog1Δ/Δ*	*hog1/hog1*	From Ching-Hsuan Lin
*C. albicans C. albicans* *C. albicans C. albicans*	*hog1Δ/HOG1* HOG1-OE SPT20-OE wt-HOG1-OE	*hog1/hog1:HOG1 spt20Δ:FRT/spt20Δ:FRT HOG1/HOG1::pAgTEF1-NAT1- AgTEF1UTR-TDH3-HOG1 hog1/hog1 SPT20/SPT20::pAgTEF1-NAT1-AgTEF1UTR-TDH3-SPT20 HOG1/HOG1::pAgTEF1-NAT1-AgTEF1UTR-TDH3-HOG1*	From Ching-Hsuan Lin This study This study This study

### Plasmid Construction

All primers and plasmids used in this study are listed in Tables 2, 3, respectively. For the creation of plasmid *pYES-CaSPT20-V5*, SC5314 genomic DNA was used as a template for *CaSPT20ResFwd* and *CaSPT20ResRev* primers, which generated a 2,678 bp DNA fragment containing *Bam*HI and *Bst*EII restriction sites, the promoter, ORF of *CaSPT20* but lacked the stop codon.

**TABLE 2:** Primers used in this study.

**Primers for strains construction**		
**Name**	**Sequence (5′-3′)**	**Usage**
*ScSPT20*DelFwd	ATGAGTGCCAATAGCCCGACAGGAAACGA TCCCCATGTATTTGGTATTCCTGTGAACGCA ACACCATCCAATATGGGTTCGCCAGGCAG TCCAGTTAATGCCGCTAGGGATAACAGGGTA	For the disruption of *ScSPT20*
*ScSPT20*DelRev	AAGTGAGAATTTTTTTTAAATAATGATGT ACTTTAATACAATATATATATATATATATATA TATATATATATATATATATAAGGAATGATAACT CTATTTGAATTCGAGCTCGTTTAAAC	
*ScKan*I deFwd	TGCCTCTTCCGACCATCAAG	For identification of the strain LCT1
*ScKan*I deRev	CCATGAGTGACGACTGAATC	
ScUpI deFwd	TGTTACCCGCTCGTGATACC	
ScUpI deRev	GGGACGAGGCAAGCTAAACA	
ScDownIdeFwd	ATACTAACGCCGCCATCCAG	
ScDownIdeRev	AACCCACTAGAGTGCATGGG	
*ScSPT20*Fwd	TATGCCCTACAACGCCCTTC	
*ScSPT20*Rev	GTGGCAAATACAGGCGCAAA	
*LacZ*Fwd	CAAGCCGTTGCTGATTCGAG	For the construction of LCT2,
*LacZ*Rev	GTGGCCTGATTCATTCCCCA	LCT3, LCT4 strains
*Ura3* Fwd	GATAGGGAGCCCTTGCATGA	
*Ura3* Rev	CGCTAAAGGCATTATCCGCC	
*CaSPT20*ResFwd	CCCGGATCCATTATATATAGCCCATAAATAAATACTG	
*CaSPT20*ResRev	CCCGGTCACCATTAGCAGGCGCATTTTTCTTCTTCTGAT	
*GAL1*F	AATATACCTCTATACTTTAACGTC	
*CaSPT20*R	GCAACAAGAAGCAAAGATTC	
*CaSPT20*F	CACTTCTGTTCACCCTCCTA	
V5-R	ATCCCTAACCCTCTCCTCGGT	
*SPT20*-OEF	AACAAAATCAGCAGTCAGTTTTTTCCAAATG GTTTAGATGACTCTTCGATTCTGGAAATGG ACGTTGAATTGAATGACAACTTAATCATAAT AAGAAATCATCAAGCTTGCCTCGTCCCC	For the construction of SPT20-OE strain
*SPT20*-OER	GTTTTCCACCCTGATTCTGAGTCAGTACTGT TGTACCATTAGATATAGAGTTTCCCACAGTT TTGGATGCAGATCCACTCAAAACTTCAGATTT TATCATATTTGAATTCAATTGTGATG	
*HOG1*-OEF	GAACACGCAACAATGCTACCGCGACTACAAAT GGTTCAATCTGGAGAGAAACTTCCACC TCAGCTAGTAACACTACTGTTTTTCTATAAACTG TTTTCACATCAAGCTTGCCTCGTCCCC	For the construction of HOG1-OE and wt-HOG1-OE strains
*HOG1*-OER	ATGCTCCCATTCCCACGGGATTTAGCTCAGTG TATCTATTGGTGATTTCAAAAACAGTC CCAAATATCTGGGTTCTTGTAAATTCTCCATC TGCAGACATATTTGAATTCAATTGTGATG	
*HOG1*-F-2	GGCATAAAAGTGTTGGTAATGGC	For colony PCR of HOG1-OE and
*NAT1*-OE-R-det	GCAGTATCATCCAAAGTAGTA	wt-HOG1-OE
*SPT20*-F-2	CTGCAACTGCACCAAGCTAT	For colony PCR of SPT20-OE
*NAT1*-R	GAAACAACAACGAAACCAGC	

**Primers for qRT-PCR**		
**Name**	**Sequence (5′-3′)**	

18S rRNA-F	CGCAAGGCTGAAACTTAAAGG	
18S rRNA-R	AGCAGACAAATCACTCCACC	
*CAT1*-F	GGCCAGTGATAAGCCAGTTG	
*CAT1*-R	TTGGATAGCAGCATCAGCAC	
*SKO1*-F	AACCACCACCACCACAAAAT	
*SKO1*-R	CACCACGCAATTCATTCACT	
*AGP2*-F	CAGTCATGGGGTTCCTGTCT	
*AGP2*-R	TACGGTTGGAACCACGATCT	
*ORF19.3661*-F	TTGTGAAGCCACTCCTGTTG	
*ORF19.3661*-R	CCAGTCGGATTAGCTTGGAA	
*HOG1*-F	GACTTGTGGTCTGTGGGTTG	
*HOG1*-R	ACATCAGCAGGAGGTGAGC	
*TGL1*-F	TATGCAAGGTTGTTCCGTCA	
*TGL1*-R	CACTGTTGCTTGCCGATCTA	
*ADH7*-F	TGAAATTGGGTGCTGATGAA	
*ADH7*-R	TGTTCAGTGGCTGGTGGTAA	
*SPT20*-F	ACAAACTACTGCTGACGGGG	
*SPT20*-R	GGAGGGTGAACAGAAGTGGG	

**TABLE 3:** Plasmids used in this study.

**Name**	**Description**	**Reference/source**
pFA6a-5FLAG-KanMX6	Amp^r^, Kan^r^	From Eishi Noguchi
pYES2.1/V5-His-TOPO	URA3, Amp^r^	From invitrogen
pYES2.1/V5-His/lacZ	URA3, Amp^r^, lacZ	From invitrogen
pYES- *CaSPT20*- V5	*CaSPT20* in pYES2.1/V5-His-TOPO	This study
pCJN542	*NAT1-TDH3* promoter	From Aaron P. Mitchell

The amplified fragment described above and plasmid *pYES2.1/V5-His/lacZ* (Invitrogen, Shanghai, China) were digested with *Bam*HI*-HF* and *Bst*EII*-HF*. The two products were purified, ligated, and the resulting plasmid was transformed to DH5α *E. coli* and colonies were selected on LB plate with 100 μg/mL ampicillin. PCR followed by sequencing were used to validate the correct insertion of *pYES-CaSPT20-V5-His*/lacZ vector (Supplementary Data).

### Generated Strains

*Saccharomyces cerevisiae* LCT1 was constructed as previously described (Marotta et al., 2013). The template plasmid pFA6a-5FLAG-KanMX6 was a gift from Eishi Noguchi (Noguchi et al., 2008; Addgene plasmid # 15983; http://n2t.net/addgene:15983; RRID: Addgene 15983). In brief, we used *ScSPT20DelFwd* and *ScSPT20DelRev* as primers and the plasmid *pFA6a-5FLAG-KanMX6* as template to amplify a 1,676 bp DNA fragment containing the kanamycin resistance gene flanked by 20 bp of *ScSPT20* 5′ and 3′ sequences. The PCR product was transferred to *S. cerevisiae* wild type strain BY4741 using a transformation method described previously by Gietz (Gietz, 2014). Transformants with the desired insert were selected on YPD media containing 200 μg/mL G418 and verified by PCR (Marotta et al., 2013). The LCT1 and BY4741 strains were transformed with the *pYES2.1/V5-His-TOPO* vector to generate LCT2 and LCT4 strains, respectively. To create strain LCT3 (*Scspt20Δ/CaSPT20*), the *pYES-CaSPT20-V5* plasmid was transformed to LCT1. All strains were verified by PCR to ensure the correct transformants were used.

The construction of overexpression strains was described previously (Nobile et al., 2008). The *NAT1-TDH3* promoter plasmid *pCJN542* (Nobile et al., 2008) was used for gene overexpression. To construct the *SPT20* overexpression strain (SPT20-OE) in *hog1*Δ/Δ mutant background, the PCR product was amplified using the plasmid *pCJN542* as template and primers *SPT20-OEF* and *SPT20-OER* (Table 2) and then transferred to *hog1*Δ/Δ mutant strain. By the same method, the PCR product generated using plasmid *pCJN542* as template for and primers *HOG1-OEF* and *HOG1-OER* (Table 2) was transferred to *spt20*Δ/Δ mutant strain to generate *HOG1* overexpression strain (HOG1-OE). Strains that underwent homologous recombination were selected on YPD+ Nourseothricin (Werner BioAgents, Jena, Germany; 400 μg/mL for SPT20-OE strain and 100 μg/mL for HOG1-OE strain) plates and the recombination events were verified by PCR with primers *SPT20-F-2* and *NAT1-R* for SPT20-OE strain, and primers *HOG1-F-2* and *NAT1-OER-det* for HOG1-OE strain, respectively. Function of this overexpression strategy was verified by real-time PCR with primers *HOG1-F* and *HOG1-R* for HOG1-OE strain, and primers *SPT20-F* and *SPT20-R* for SPT20-OE strain, respectively.

### Sensitivity Assays

Sensitivity to a range of stresses was evaluated using a solid media assay. All investigational strains were grown to mid-log phase under suitable growth conditions and collected by centrifugation. The pellets were suspended in YPD at 2.5 × 10^7^ cells/mL. Ten-fold serial dilutions from 2.5 × 10^7^ to 2.5 × 10^3^ of all strains were prepared, and 4 μL of each of strain dilutions was spotted onto the agar plates with integrated stimuli. Cells were incubated at 30°C for 48 h and then observed for growth differences.

### RNA Isolation and qRT-PCR Analysis

The *C. albicans* strains SC5314, *Caspt20Δ/Δ*, *Caspt20Δ/SPT20* were cultured to logarithmic phase and diluted to OD_600_ = 0.2. The cultures were incubated at 30°C with shaking for 4 h. 5 × 10^7^ cells were counted with a hemocytometer and then collected by centrifugation. After being washed twice with sterile PBS, the pellets were subjected to 1 M NaCl in YPD, while the control group was added to an equal volume of YPD medium. All the cultures were grown at 30°C with shaking for an additional 30 min. After treatment, cells were collected by gentle centrifugation, and total RNA was extracted using an RNeasy Mini Kit (Qiagen, Shanghai, China) according to the manufacturer’s protocol. The concentration, purity, and integrity of RNA were checked by Nanodrop spectrophotometer. Generally, RNA samples with an A_260_/A_280_ ratio between 1.9 and 2.1 were used for further interrogation.

In order to remove potential genomic DNA contamination and synthesize cDNA, we used PrimeScript^TM^ RT reagent Kit with gDNA Eraser (Perfect Real Time; TaKaRa, Dalian, China) following the manufacturer’s protocol. Real time reactions were prepared using TB Green^TM^ Premix Ex Taq II^TM^ Kit (Tli RNaseH Plus; TaKaRa, Dalian, China), and quantitative PCR experiments were conducted in a LightCycler480 System (Roche, Switzerland). Transcript levels were normalized against 18S rRNA expression (used as an internal control of gene expression). The gene expression changes were measured in 2^–ΔΔCt^ method. Fold changes of target genes in the *spt20* mutant and reconstituted strains were normalized to the untreated wild type strain.

### Intracellular Glycerol Assays

The *C. albicans* production of intracellular glycerol were measured as previously described (Ene et al., 2015). In brief, *C. albicans* strains SC5314, *Caspt20Δ/Δ*, and *Caspt20Δ/SPT20* were grown overnight. An aliquot of 5 × 10^7^ cells were treated with 1 M NaCl in YPD for 30 min. Subsequently, the intracellular glycerol levels were measured using the Free Glycerol Reagent (Sigma-Aldrich, Shanghai, China) according to the manufacturer’s protocol.

### Western Blotting

*Candida albicans* wild type strain SC5314 and null mutant strain *Caspt20Δ/Δ* were grown to mid-exponential phase in YPD at 30°C with shaking. Cells were exposed to hyperosmotic stress for a designated period of time by adding 5 M NaCl stock solution to YPD medium to achieve a final concentration of 2 M NaCl. As a control, equal volume of YPD was added instead of NaCl. Following treatment, *C. albicans* cells were collected and the pellets were washed twice with sterile PBS. To extract protein, pellets were suspended in 200 μL RIPA lysis buffer containing Protease Inhibitor Cocktail (Roche, Shanghai, China), and an equal volume of acid-washed glass beads (Sigma-Aldrich, shanghai, China) was added. The cells were vigorously vortexed for 1 min to mechanically disrupt cell walls then transferred to ice for 1 min, and vortex and chill process repeated six more times. Cell extracts were separated from whole cell debris and glass beads by applying centrifugation at 13,000 rpm at 4°C for 10 min.

The protein concentrations were determined using a Pierce BCA Protein Assay Kit (Thermo Fisher Scientific, Shanghai, China). Equal quantities of protein (40 μg) were loaded onto a 10% gel, analyzed by SDS-PAGE, and then transferred to PVDF membranes. Anti-phospho-P38 antibody (Cell Signaling Technology, Shanghai, China) was used to detect the phosphorylated form of Hog1 (Smith et al., 2004). Total Hog1 level was detected by Hog1 (D-3) antibody (Santa Cruz Biotechnology, Shanghai, China). β-anti-actin antibody (GeneTex, Shenzhen, China) was used as the loading control (Deng and Lin, 2018).

### Statistical Analysis

All experiments were performed at least twice as independent replicates. Data were analyzed using SPSS software. Student’s *t*-test and the analyses of variance (ANOVA) were used to determine statistical significance. A *P*-value < 0.05 was considered statistically significant.

## Results

### Conservation of CaSPT20

We have previously reported that *SPT20* was involved in regulating virulence and stress responses in *C. albicans* (Tan et al., 2014). However, little is known about the underlying molecular mechanisms. The amino acid sequence alignment showed there are conserved functional domains between CaSpt20 and ScSpt20 (Supplementary Figure 4). With the hypothesis that *C*. *albicans SPT20* could be functionally conserved with *Saccharomyces cerevisiae*, we endeavored to determine if *CaSPT20* could restore defects in *ScSPT20* mutant strains. To this end, we constructed *S. cerevisiae* strains LCT1 (*Scspt20*Δ), LCT2 (*pYES2.1/V5-His-TOPO* in the LCT1 background), LCT3 (*pYES2.1/V5-His-TOPO-CaSPT20* in the LCT1 background) and LCT4 (*pYES2.1/V5-His-TOPO* in the background of the wild type strain BY4741), then performed a series of functional complement assays.

**FIGURE 1:**
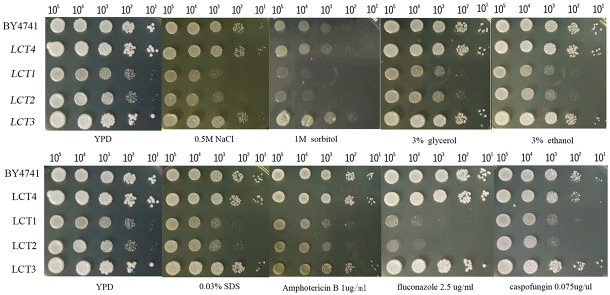
The introduction of *CaSPT20* can restore sensitivity to stresses and antifungal agents caused by the loss of *ScSPT20*. LCT1 (*Scspt20Δ*), LCT2 (*Scspt20Δ*+pYES2.1/V5-His-TOPO), LCT3 (*Scspt20Δ*+*pYES- CaSPT20-V5*), and LCT4(BY4741+pYES2.1/V5-His-TOPO) were constructed in the background of *S. cerevisiae* BY4741 and grown at 30°C for 48 h. The experiment was repeated on 3 independent occasions.

The strains were grown on YPD agar plates supplemented with hyperosmotic stressors (NaCl, sorbitol, and glycerol), ethanol stress, cell wall stress agent SDS, or antifungal agents (amphotericin B, fluconazole, and caspofungin), which directly perturb cell membrane component ergosterol synthesis or FKS required for cell wall synthesis. After cultivation for 48 h, cell growth was observed under the applied stress conditions. The introduction of plasmid *pYES2.1/V5-His-TOPO* had no influence on cell growth, as seen when comparing growth of LCT4 to BY4741, and LCT2 to LCT1. Deletion of *SPT20* impaired normal cell growth of *S. cerevisiae*, which was in agreement with the results reported by Roberts and Winston (Roberts and Winston, 1996), but growth retardation was exacerbated when cells were associated with the tested hyperosmotic stressors and cell membrane targeting antifungal agent fluconazole. Notably, the cell growth of *Scspt20Δ* mutant was rescued with complementation of *CaSPT20*, suggesting that *SPT20* is required for the normal cell growth under extracellular osmolarity and cell membrane stressor exposure. However, the decrease in resistance to the other stresses (such as ethanol, SDS, amphotericin B, and caspofungin) seen in *Scspt20*Δ cells largely matches the decreased growth seen in the BY4741 (Figure 1), indicating that the growth defects of *Scspt20*Δ cells in these stresses may not be due to the deletion of *SPT20*. Importantly, complementation of *CaSPT20* restores the growth of *Scspt20Δ* to the wild type levels, supporting that the function of *SPT20* is conserved between *C. albicans* and *S. cerevisiae*.

### Hog1 Phosphorylation Is Reduced by Deletion of CaSPT20

The data demonstrates that Spt20 plays a conserved role in protecting cells from osmotic stress. A well-known contributor in protecting fungi from osmotic stress is the Hog1 pathway (Brewster and Gustin, 2014). When *C. albicans* is exposed to high osmolarity, Hog1 is phosphorylated and then induces target gene expression to adapt to osmotic stress (Smith et al., 2004; Day et al., 2017). In other words, phosphorylation of Hog1 is the essential step for *C. albicans* to survive during high osmotic challenge. To test if *CaSPT20* affects Hog1 responses to osmotic stress, we extracted protein from the indicated strains subjected to 2 M NaCl in YPD for various time periods, and then performed western blotting. Specific antibodies were used to detect the levels of total Hog1 and phospho-Hog1, respectively. In this assay, β-actin antibody was used as a loading control.

**FIGURE 2:**
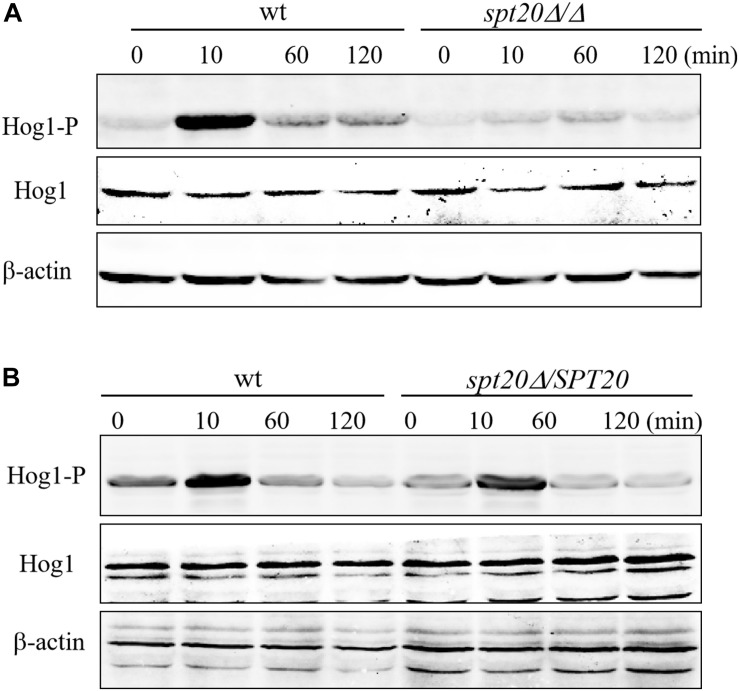
*SPT20* affects the phosphorylation level of Hog1 protein. Cells were exposed to 2 M NaCl for the indicated time. phosphorylated Hog1 (Hog1-P) and total Hog1 (Hog1) levels of wild type, *SPT20* null mutant **(A)**, and *SPT20* reconstitution strain **(B)** were detected by western blotting with specific antibodies. The beta actin antibody was used as the loading control. This experiment was repeated three times independently.

As reported previously (Alonso-Monge et al., 2003; Smith et al., 2004), Hog1 phosphorylation was induced by osmotic treatment. Hog1 phosphorylation peaked after 10 min under high osmotic stimulation in wild type SC5314, however, *Caspt20Δ/Δ* failed to have the same level of Hog1 phosphorylation after 10 min of osmotic treatment. Indeed, only a very slight increase in phosphorylated Hog1 was observed after 60 min of stimulation (Figure 2A). In stark contrast, reconstitution of *SPT20* restored phospho-Hog1 to wild type levels (Figure 2B). In addition, the level of total Hog1 transcription in these three strains remained constant, which was in accord with what Enjalbert et al. reported (Enjalbert et al., 2006), indicating that Hog1 phosphorylation occurs independent of total Hog1 expression levels. Thus, it appears that *SPT20* correlated with the phosphorylation of Hog1. We then further explored the effects that deletion of *SPT20* has on Hog1 responses and the *C*. *albicans* phenotype.

### CaSPT20 Affects Expression of Hog1-Dependent Osmotic Stress Response Genes

**FIGURE 3:**
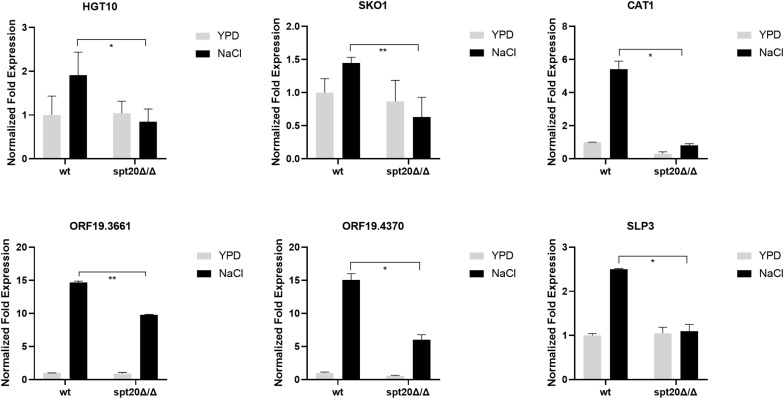
Expression levels of Hog1-dependent osmotic stress response genes changed significantly when *SPT20* was deleted in *C. albicans*. qRT-PCR expression analysis of *C. albicans* Hog1-dependent osmotic stress response genes in the wild type strain, *spt20*Δ/Δ and *spt20*Δ*/SPT20.* Transcript levels were normalized to *C. albicans* 18S rRNA expression and fold changes between strains were normalized to untreated wild type which was adjusted to a value of 1. All genes were analyzed in triplicate. Error bar represents Mean ± SD. *P < 0.5, **P < 0.01.

As shown above, the level of phosphorylated Hog1 in *Caspt20Δ/Δ* was much less than what was seen in the wild type strain, suggesting that *SPT20* affected the level of phosphorylated Hog1, which prompted us to investigate whether the expression levels of Hog1-dependent osmotic stress response genes were affected by the loss of *SPT20*. To this end, we measured the expression of Hog1-dependent osmotic stress response genes. A panel of genes was assembled for interrogation as the previous work did, which reported the expression levels of *HGT10* (encoding a glycerol permease involved in active glycerol uptake), *SKO1* (encoding a transcriptional factor binding to promoters to relieve osmotic stress), *CAT1* (one of core stress genes, encoding a key antioxidant enzyme), *ORF19.4370* (predicted ORF), *ORF19.3661* (encoding a putative deubiquitinating enzyme) and *SLP3* (encoding a putative cation conductance protein) were induced in a Hog1-dependent manner (Enjalbert et al., 2006; Marotta et al., 2013). As showed in Figure 3, these Hog1-dependent osmotic stress response genes were significantly upregulated in wild type cells encountering hyperosmolarity, while the expression of *HGT10*, *SKO1*, *CAT1*, and *SLP3* were not induced when *SPT20* was deleted. Although the transcript of *ORF19.3661* and *ORF19.4370* in *Caspt20Δ/Δ* was induced in the presence of 1 M NaCl, it still did not reach the level observed in the wild type (*ORF19.3661*, *P* = 0.007; *ORF19.4370*, *P* = 0.011). Notably, the transcriptional changes caused by the deletion of *SPT20* gene in *C. albicans* were in accordance with that caused by the loss of *HOG1* gene, which also exhibited reduced expression of *HGT10*, *SKO1*, *CAT1*, *ORF19.4370*, *ORF19.3661*, and *SLP3* in the *hog1Δ/Δ* mutant strain during 1 M NaCl stimulation (Marotta et al., 2013). Taken together, we can conclude that *SPT20* plays a role in appropriate expression of Hog1-dependent osmotic stress response genes.

### The Cell Growth Defect of Caspt20Δ/Δ Is Rescued by HOG1 Overexpression Under Osmotic Stress

**FIGURE 4:**
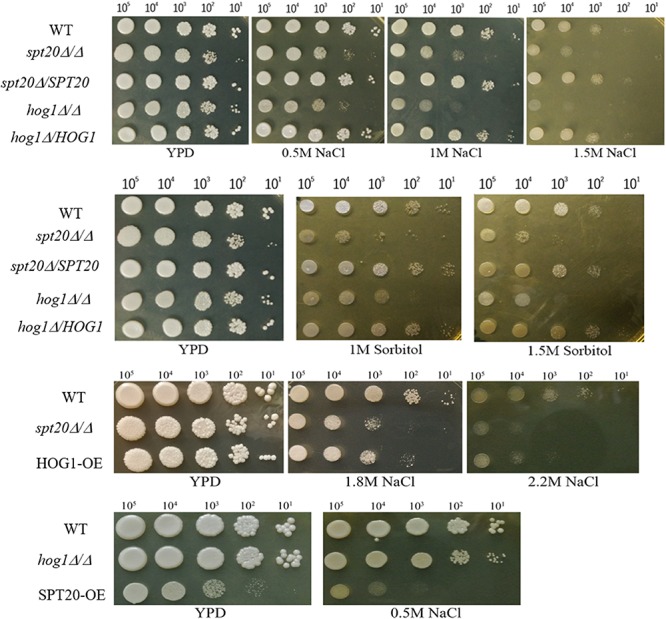
The cell growth defect of *Caspt20Δ/Δ* is similar to that of *Cahog1Δ/Δ* and is rescued by *HOG1* overexpression. Ten-fold serial dilutions of strains were spotted on appropriate plates to evaluate the cell growth defects. Cells were grown at 30°C and then photographed. The experiment was repeated on three independent occasions.

The Hog1-MAPK pathway has been reported to be involved in osmoadaptation, take part in resistance to oxidative stress, and also play a role in morphogenesis changes as well as cell wall biosynthesis (Monge et al., 2006). As we demonstrated earlier, the ability to overcome hyperosmotic stress was impaired in *Caspt20*Δ/Δ, suggesting *CaSPT20* really plays a role in osmostress responses (Tan et al., 2014). To evaluate if *SPT20* responds to osmotic stress in cooperation with the Hog1 pathway, cell growth of strains SC5314, *Caspt20Δ/Δ*, *Caspt20Δ/SPT20*, *Cahog1Δ/Δ* and *Cahog1Δ/HOG1* were examined during exposure to external hyperosmolarity, comparing growth defects between *Caspt20Δ/Δ* and *Cahog1Δ/Δ* strains. As expected, the knockout of *CaSPT20* or *CaHOG1* both led to impaired cell growth in hyperosmotic conditions imposed by NaCl or sorbitol, and for each, growth was restored to wild type levels when *CaSPT20* and *CaHOG1* were reconstituted, respectively (Figure 4). This result, along with the affected phosphorylation level of Hog1 and the expression of Hog1-dependent osmotic stress response genes, suggest a link between *SPT20* and Hog1-MAPK pathway in *C. albicans* osmoadaptation. Thus, we hypothesized that *SPT20* regulated the Hog1-MAPK pathway to respond to external osmotic stress. In order to evaluate this hypothesis, we constructed the *HOG1* overexpression strain in the *Caspt20*Δ/Δ mutant background (HOG1-OE) and in the wild type background (wt-HOG1-OE), and *SPT20* overexpression strain in the *Cahog1*Δ/Δ mutant background (SPT20-OE). The gene overexpression was verified by quantitative reverse transcription-PCR analysis. In addition, the basal levels of phosphorylated Hog1 and total Hog1 in the HOG1 overexpression strains were significantly increased when compared with wild type (Supplementary Figure 1). We compared the cell growth of strains SC5314, *Caspt20*Δ/Δ, HOG1-OE, *Cahog1*Δ/Δ, and SPT20-OE, which were treated with NaCl. As illustrated in Figure 4, we observed that *HOG1* overexpression can partially rescue the growth defect caused by *SPT20* deletion when cells were exposed to a series of high osmotic stress (YPD plate supplements with 1.8 M NaCl, or 2.2 M NaCl), while overexpressing *SPT20* did not confer the ability to resist hyperosmotic stress to the *hog1*Δ/Δ strain. Furthermore, in order to investigate whether there exists a Spt20-independent manner contributing to the increasing resistance of HOG1-OE strain, we observed the cell growth of wt-HOG1-OE strain, and found that overexpressing *HOG1* did not enhance the osmotic tolerance of wild type cells (Supplementary Figure 2). These results indicate that *SPT20* influences Hog1 during the *C. albicans* response to osmotic stress.

### CaSPT20 Regulates Glycerol Accumulation in *C. albicans*

**FIGURE 5:**
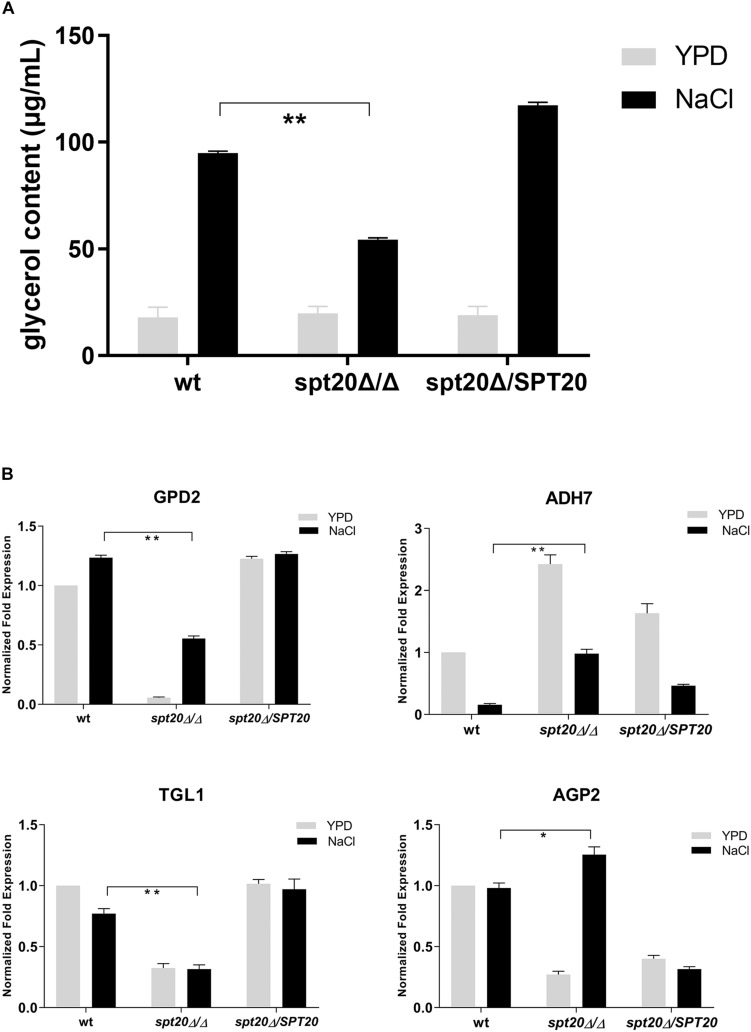
*CaSPT20* regulates intracellular glycerol accumulation under hyperosmotic condition. **(A)** *CaSPT20* has an important effect on intracellular glycerol content under hyperosmotic condition. 5 × 10^7^ cells with or without treatment (1 M NaCl or YPD) were grown at 30°C for an additional 30 min and then used for glycerol measurement. The ability of *spt20*Δ/Δ strain to accumulate cellular glycerol under osmotic stress was impaired. **(B)** *SPT20* affects the expression of genes involved in glycerol accumulation. qRT-PCR analysis was used to measure the expression of genes involved in glycerol accumulation following exposure to 1 M NaCl. The following *C*. *albicans* strains were evaluated: wild type SC5314, *spt20*Δ/Δ, and *spt20*Δ*/SPT20* and genes were analyzed in triplicate. Expression levels were normalized to 18S rRNA. Fold changes between strains were normalized to untreated wild type, which was adjusted to a value of 1. Three independent biological replicates were conducted. Error bar represents Mean ± SD. *P < 0.01, **P < 0.001.

To investigate the correlation between *CaSPT20* and Hog1-MAPK pathway in hyperosmotic stress response, we measured the intracellular glycerol accumulation of strains SC5314, *Caspt20Δ/Δ*, and *Caspt20Δ/SPT20* after exposure to 1 M NaCl for 30 min. Our results showed the basal glycerol contents of these three strains were almost the same, and they all increased strikingly under hyperosmotic condition. However, the ability to accumulate intracellular glycerol was impaired in *Caspt20Δ/Δ* strain (*P* < 0.001) (Figure 5A).

Glycerol biosynthesis is catalyzed by glycerol-3-phosphate dehydrogenase (encoded by *GPD1/GPD2*) and glycerol-3-phosphatase (encoded by *RHR2*) (Hohmann, 2002; Fan et al., 2005). Meanwhile, the increased cellular glycerol concentration is also forced by regulated activities of triacylglycerol lipases (encoded by *TGL1/TGL2*) (Wei et al., 2009), alcohol dehydrogenase (encoded by ADH) (Blomberg and Adler, 1989), and amino acid permease (encoded by *AGP2*) (Marotta et al., 2013). To further examine the Spt20 influence on cellular glycerol, we examined expression of these genes involved in glycerol accumulation. *GPD2*, which was reported to increase to a greater extent than *GPD1* and *RHR2* levels in response to osmostress (Smith et al., 2004; Enjalbert et al., 2006; Jacobsen et al., 2018), was suppressed when *CaSPT20* was knocked out. Although *GPD2* was induced in the presence of 1 M NaCl, its expression still did not reach the level observed in the wild type (*P* < 0.001). In contrast, *ADH7* was significantly induced in the *Caspt20Δ/Δ* mutant, both in the absence and presence of osmotic stress (6-fold in the presence of 1 M NaCl compared to wild type; *P* < 0.001). *TGL1* was under-expressed in *Caspt20Δ/Δ* after exposure to osmotic stress (0.31 for the mutant versus 0.76 for wt strain; *P* < 0.001). When compared to wild type, *AGP2*, a gene involved in membrane permeability, experienced reduced expression in the absence of salt exposure in the *Caspt20Δ/Δ* strain, but following osmotic stress treatment, it significantly elevated and was 1.25-fold higher than the expression found in the wild type strain (*P* = 0.002) (Figure 5B). The decreased expression of *GPD2* can reduce the accumulation of intercellular glycerol directly. The expression of *ADH7* can reduce the yield of NADH, then lead to the decreasing production of glycerol (Blomberg and Adler, 1989). Meanwhile, the reduced transcript level of *TGL1* blocks the process of transforming triglycerides to glycerol (Jandrositz et al., 2005; Wei et al., 2009), while the upregulation of *AGP2* can cause increasing membrane permeability, so that the intracellular glycerol can spread to extracellular environments. Taken together, these findings suggest that *CaSPT20* participates in the process of glycerol accumulation, since *GPD2*, *ADH7*, *TGL1* and *AGP2* expression were sharply affected when *SPT20* was deleted from *C. albicans*.

## Discussion

Osmoregulation by homeostatic mechanisms is crucial in *C. albicans* in order to keep appropriate cell volume, turgor, as well as a suitable intracellular environment for all kinds of biochemical reactions (Hohmann et al., 2007; Fuchs and Mylonakis, 2009). In this paper, we show that CaSpt20 has functional similarity with ScSpt20 and can be used to reconstitute a mutation in the homologous gene. The increased sensitivity of *Caspt20Δ/Δ* to hyperosmolarity is due to its reduced phosphorylation levels of Hog1, thereby causing downregulation of osmotic stress response genes and decrease in glycerol accumulation, suggesting that *SPT20* is involved in resistance to high osmolarity. These findings give us new insight into the role of *SPT20* in *C. albicans* response to osmotic stress, and indicate a new relationship between Spt20 and Hog1.

As an indispensable component of the SAGA complex, *SPT20* has gained enough attention on its function. It was reported that Spt20 was involved in endoplasmic reticulum stress response in human (Nagy et al., 2009), hypoxic response (Hickman et al., 2011) and the functional interaction between other SAGA components and TBP in yeast (Roberts and Winston, 1997), and the calcineurin-mediated Cl^–^ homeostasis in *Schizosaccharomyces pombe* (Zhou et al., 2013). Furthermore, *SPT20* is required for normal cell growth (Roberts and Winston, 1996) and is essential for yeast survival at high osmolarity (Zapater et al., 2007). Here, we demonstrated that knockout of *ScSPT20* caused significantly further growth defects associated with the tested hyperosmotic stressors (NaCl and sorbitol) compared to a wild type control exposed to the same conditions. Additionally, the ability of *Caspt20Δ/Δ* mutant strain to resist hyperosmolarity was greatly impaired (Figure 4). These results suggested that *SPT20* is required for the normal cell growth under osmotic condition, which was in accord with the previous work that reported *SPT20* is essential for yeast survival at high osmolarity (Zapater et al., 2007). The similar phenotypes between *Scspt20Δ* and *Caspt20Δ/Δ* mutant strain, and increasing resistance to osmotic stress due to the complement of *CaSPT20*, supported that the function of *SPT20* was conserved.

The Hog1-MAPK pathway is critical for *C. albicans* to respond to osmotic stress. Hog1, the core component in this pathway, has a strong functional preservation from yeast to mammals (Sheikh-Hamad and Gustin, 2004), and its rapid phosphorylation is an essential step in osmotic toleration (Brewster and Gustin, 2014). Our findings suggest that Spt20 regulates Hog1 activation in *C. albicans* response to hyperosmotic stress. The evidences are the following. First, the cell growth defect of *Caspt20Δ/Δ* was similar to that of *Cahog1Δ/Δ* (Figure 4). Second, the phosphorylation level of Hog1 was significantly decreased because of the absence of *SPT20* (Figure 2). Our western blotting result showed that, as reported previously (Smith et al., 2004), Hog1 was rapidly but transiently phosphorylated during *C. albicans* salt exposure. However, phosphorylation levels were comparably lower in *Caspt20*Δ/Δ, suggesting that *CaSPT20* affected the process of Hog1 phosphorylation. Hog1 phosphorylation is a dynamic event (Alonso-Monge et al., 2003; Smith et al., 2004). The kinetics of phosphorylation were different in these two strains: wild type strain peaked at about 10 min after exposure to stress, while the mutant strain peaked at about 60 min. However, in both wild type and mutant strains, the peak levels of phospho-Hog1 were diminished over time. Third, overexpressing *HOG1* in the *spt20Δ/Δ* mutant background can partially rescue the growth defect when *spt20Δ/Δ* mutant strain was exposed to osmotic stress, while overexpressing *SPT20* in the *hog1*Δ/Δ mutant background was not able to restore its ability to respond to hyperosmolarity (Figure 4). Meanwhile, the HOG1-OE strain demonstrated higher basal level of phosphorylated Hog1 and total Hog1 protein than wild type and *spt20Δ/Δ* mutant strain (Figure 2 and Supplementary Figure 1B), which may contribute to tolerate its osmostress, since *C. albicans* regulates the phosphorylation of Hog1 to respond to hyperosmolarity (Smith et al., 2004). Though the basal phosphorylated Hog1 level of wt-HOG1-OE strain was enhanced as well, overexpression of *HOG1* did not increase the resistance to osmolarity in wild type. Our working hypothesis is that the phospho-Hog1 level in wild type may be similar to that in wt-HOG1-OE strain under osmotic exposure which leads to similar cell growth. Additionally, overexpression *HOG1* did not revert the growth defect of *Caspt20Δ/Δ* mutant strain to a level comparable to the wild type, suggesting that besides the Hog1-MAPK pathway, there may exist another mechanism that accounts for *SPT20* response to osmotic stress. Strikingly, *SPT20* overexpression in the *hog1Δ/Δ* mutant background reduced the cell growth in the YPD plate. This phenotype was not due to the changes in the shape of fungal cells, since the SPT20-OE strain cells grew as unicellular yeast and the shape was similar to that of wild type and *hog1Δ/Δ* mutant strain (Supplementary Figure 3). *SPT20* is crucial for the structural integrity of SAGA complex (Grant et al., 1997; Sterner et al., 1999), thus, overexpressing *SPT20* may change the structure of SAGA complex, which would hamper the normal gene expression and then impair normal cell growth.

We noticed that, compared to wild type, the transcript level of genes involved in glycerol accumulation was either reduced or induced in the *Caspt20Δ/Δ* mutant strain (Figure 5B). However, the changing patterns of these genes were similar to that of wild type. When *C. albicans* cells were subjected to osmotic stress, the activation of the Hog1-MAPK pathway can regulate the synthesis and accumulation of glycerol (San José et al., 1996; Monge et al., 2006), along with up-regulation of genes contributing to increase the intercellular glycerol levels and down-regulation of genes contributing to reduce the glycerol levels. Although the magnitude of Hog1 activation was significantly decreased in *Caspt20Δ/Δ* mutant strain (Figure 2A), the reduced phosphorylated Hog1 can still induce or repress the related gene expression to cope with osmolarity. Strikingly, the fold induction of *GPD2* in *Caspt20Δ/Δ* mutant strain is greater than that in wild type. We hypothesized that the repressed expression of *ADH7* and *TGL1*, together with the up-regulation of *AGP2* contribute to the decrease of intracellular glycerol, which may in turn lead to a greater fold induction of *GPD2* in *Caspt20Δ/Δ* mutant strain response to osmotic stress. Furthermore, in contrast to the induction of *GPD2* expression reported previously (Enjalbert et al., 2006; Marotta et al., 2013), there was no increase in *GPD2* expression in wild type cells when osmotic stress was imposed. However, the transcript level of *GPD2* is dynamic and related to the incubation time upon osmotic stress (Enjalbert et al., 2006) and the *C. albicans* wild type strains used in these studies were different, thus we speculated that the incubation time and the wild type strains were associated with the different *GPD2* expression.

Our study has limitations that need to be taken into account and addressed in the future. First, although the growth defects of *Scspt20Δ* were rescued with complementation of *CaSPT20* (Figure 1), *C. albicans SPT20* gene was not codon optimized prior to expression in *S. cerevisiae*, which should be noted as a limitation in our study because it may lead to mistranslation. Also, in future work, we plan to evaluate a *Caspt20Δ/hog1Δ* double mutant strain to further assess genetic epistasis between *SPT20* and *HOG1*.

## Conclusion

**FIGURE 6:**
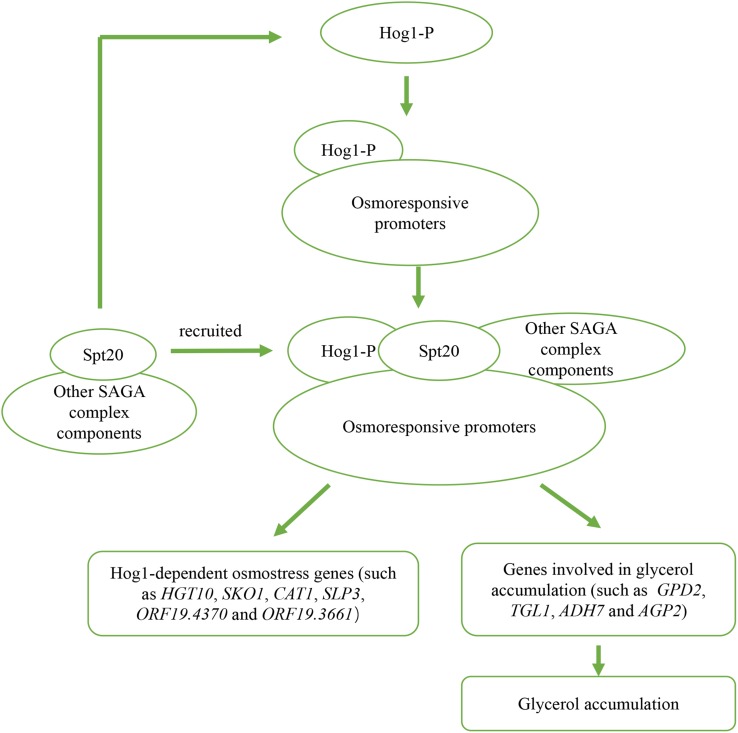
Outline of two potential mechanisms of Spt20 in *C. albicans* response to osmotic stress. Previous work found that when cells are subjected to high osmolarity, Hog1 is activated and bind to osmostress promoters, then recruits SAGA complex components to induce expression of Hog1-dependent osmostress response genes and genes involved in glycerol accumulation. In our study, we found that Spt20 regulates the phosphorylation of Hog1 in response to osmotic stress.

In conclusion, we confirm that *SPT20* is functionally conserved between *S. cerevisiae* and *C. albicans*, and report that *SPT20* plays a critical role in *C. albicans* response to hyperosmotic stress through regulating Hog1-MAPK pathway, through both expression and phosphorylation (Figure 6). The reduced Hog1 phosphorylation in *Caspt20*Δ/Δ mutant can explain its high sensitivity to osmotic stress, indicating a relationship between Spt20 and Hog1 in the response to altered osmotic conditions.

## Data Availability Statement

All datasets generated for this study are included in the article/Supplementary Material.

## Author Contributions

RC, LW, BF, XT, and EM designed the study. LW, RC, SL, and QW performed the experiments. SL, HW, and LL helped to analyze the data. LW, BF, XT, and EM wrote the manuscript. XT contributed to reagents and materials, and supervised the project.

## Conflict of Interest

The authors declare that the research was conducted in the absence of any commercial or financial relationships that could be construed as a potential conflict of interest.
