# Supplementary material for: SPT20 Regulates the Hog1-MAPK Pathway and Is Involved in Candida albicans Response to Hyperosmotic Stress
Source: Front Microbiol. 2020 Feb 21;11:213. doi: 10.3389/fmicb.2020.00213 (PMC7047840; doi:10.3389/fmicb.2020.00213)
Supplement: Supplementary file 1 [file Data_Sheet_1.docx]

Supplementary Material

# Supplementary Data

## Data 1. The sequence of plasmid *pYES-CaSPT20-V5* (8536bp).

ACGGATTAGAAGCCGCCGAGCGGGTGACAGCCCTCCGAAGGAAGACTCTCCTCCGTGCGTCCTCGTCTTCACCGGTCGCGTTCCTGAAACGCAGATGTGCCTCGCGCCGCACTGCTCCGAACAATAAAGATTCTACAATACTAGCTTTTATGGTTATGAAGAGGAAAAATTGGCAGTAACCTGGCCCCACAAACCTTCAAATGAACGAATCAAATTAACAACCATAGGATGATAATGCGATTAGTTTTTTAGCCTTATTTCTGGGGTAATTAATCAGCGAAGCGATGATTTTTGATCTATTAACAGATATATAAATGCAAAAACTGCATAACCACTTTAACTAATACTTTCAACATTTTCGGTTTGTATTACTTCTTATTCAAATGTAATAAAAGTATCAACAAAAAATTGTTAATATACCTCTATACTTTAACGTCAAGGAGAAAAAACCCCGGATCGGACTACTAGCAGCTGTAATACGACTCACTATAGGGAATATTAAGCTCGCCCTTGGATCCATTATATATAGCCCATAAATAAATACTGTTATCAAAGTAATTTTTATTGTAAGAAGTTCCTCTAATAACTTACCAAACTGACGCAGATTAGATTTTGTATTCTGGAAGATAGATTTTTTTTTCGAGACTCTTTTTTTTTTTCGTTGCAAGATACATGTATTCTACAAATAAACGAAACACAACAACTCCAAATACAAGTGGTGCATTTGACTACTAGAATATTGATCCAATCAATCACTGATTACGAAGTTGTTATCTTATTTTCAAGATATAATCATTCAGCTGAGTTTTTTTTTTAGAATTTCATTAAAGTTAGAAGACACAATTGTGAAAAAAGAGCTTTAGTCAATCATAAACAAATAGTCATATTAAATAGATTCATTAAGACTACCTTTGACATTACACTATGATAAAATCTGAAGTTTTGAGTGGATCTGCATCCAAAACTGTGGGAAACTCTATATCTAATGGTACAACAGTACTGACTCAGAATCAGGGTGGAAAACAGAATCTAATCCGTCAGCAGCAGCAGCAGCAGCAACAACAACAACAACAACAACAACAGCAGCAGCAGCAGCAACTCAAACACAGAACTGCTCTTCAAAATTACCATTTTGCCTCAACTTCGGAAGAGATCTTAAAAAAATACTCCAAATATCCAGCATCCATGAGTTTACATATTTTTGAAACGCATTACAGATTTAACAATTCTCAAGATTCCCAAGTAATTCCCAAGGATTCGCCCATGATTAAAGATTTTATGAAGCATGTTTTGAAAGAGCAAATTCCCGTTGAAATGTGTGAATTAATCAAAGATTTTGCTATTAGACCGTATGATGGATGCATAATATTACAAGTTTATGATCACAGAAATATGGTTAAAACAGCTGTTTTACAGAACAGATCAACATCGTCTCCATCCAAAGATCAAGACAAAAAGCAACAAATGGTTGTTTCAAAGCCCCGGACATATAGAACTTTGTTGAGACCAACTTCCCTTTCTATTTATTACGATTTACTTTATCATACAGACTCGGCACTCCATCGATTCACTGATTATTTATCTTTACAAATGGAGTCTGAGATTTTGACAGCAACAAATAGAGAGTTAAATTTATCAGTTCCTTTGAATCCTTATAATTACGATCACCTTAGACCTGAACCTGAACCTTCTGGCGATATGTCTCAAAACAGTGACGAAGAGATTGACCAAGTTAAATTTCAACACAGAGATGCAGTTGAACAACCTCGTCGTAAAATTCATCAAGACGAAATGGTGTTGCACAAATCTTCGGAATACGAAGAACTTATGTTGCTATTATCTAACAAACATAAACGTCCAGATGATTGTCTGGATAAACGACTCGTGGTTGTAAGTACTTCGGCATTGCCAGGTTCTTCGCTAACTGGAACCTCTAATACATCAACAGGAACAGCGGCTGCAACACCTACACCTACAAGTACAACTGCATCAGCTGCTTCTCAGTCTTCAAAACAAACTACTGCTGACGGGGCATCGACAACGGATACCAAAGGCAAGAAAGGAGACAAAGCAAATGGCACCAGTGCAAATCAAGCACTGGCATCGCCCACTTCTGTTCACCCTCCTATTCCGACCATACCACAAAATCTGGTTAGAGCTACTGGACAGTTCATGAGGTTGAGATTAATAGAGGAAATAAGAAAGAAAAGAGAACTTGAGAAGTTGCAACAAGAAGCAAAGATTCAGGCCCAGGCAAATGCCATTCAAAGTGATCTGGTACCACCCAGTCAACAATTGGCTCCTCAGAATACCATAAACTCGCCTGTTGTCACTGAACCGACAAAGCGTTCATCTCCAGCTGCTCAGAATCAACCTGCGCCCAAAAGAGCGAAGAAAGAGACAAAAAAACAATTACAAGCCAAAGCACAAGCTCAAGCAAAAGCCCAGGCACAAGCAAAAGCAAAAGTACAAACCGAAACTCCAGGCCCGACTCCTGTCCAGGGACAAAATTCATTGTCGGGTCCACTGGTGGTTAATGGGTCTAATGTTACAAGTACAGCAAATACACCCACGATGAATAATCAAAGTAGTCTTCAACAACCTTCCCAAGCACTGCAACCGCAAATTGGAAGTTTGAATAACAATCAACAGTCACAATCACAGCAACAACAACAGAAAAATCCCCAACAGACTCTTCAGCAACAGCAGCAACAACAGATATTCCAGAATTCATTGACACCTGAAGAACAAAAAGTGTATAAGCAAATCCAACAGAATATGGCTACACTTGCAATGATGGGCCAATCTGGAGTTACCCCTACGGGCCAACAATTGACACCACAACAAAAACAACAAGCAATTCAACAAGCGAAGAACTTACAACAGCAATTATTTCAACGATTCCCGCTTTATTTTCAACGAATGAAACAGTTGCAGCTAATTCACCAAAAGAGAAGATTACAGCAGCAGCAGCAACAACAGCAACAACAACAACAACAGCAAGCCTCTAATTCCTCCGGTGGTAATCAAAATAATTCATCGAATCAAGCCGCCCCCCAAACTCAACAATTTAATCAAGCATTGCCATCTACGTCAGAGCAGTCCAAACCTGTTACGACATCGCCAGAAGTTAAAAAGAAAAGAACCTATCAGAAGAAGAAAAATGCGCCTGCTAATGGTCACCCATTCGAAGGTAAGCCTATCCCTAACCCTCTCCTCGGTCTCGATTCTACGCGTACCGGTCATCATCACCATCACCATTGAGTTTCTAGAGGGCCGCATCATGTAATTAGTTATGTCACGCTTACATTCACGCCCTCCCCCCACATCCGCTCTAACCGAAAAGGAAGGAGTTAGACAACCTGAAGTCTAGGTCCCTATTTATTTTTTTATAGTTATGTTAGTATTAAGAACGTTATTTATATTTCAAATTTTTCTTTTTTTTCTGTACAGACGCGTGTACGCATGTAACATTATACTGAAAACCTTGCTTGAGAAGGTTTTGGGACGCTCGAAGGCTTTAATTTGCAAGCTGCGGCCCTGCATTAATGAATCGGCCAACGCGCGGGGAGAGGCGGTTTGCGTATTGGGCGCTCTTCCGCTTCCTCGCTCACTGACTCGCTGCGCTCGGTCGTTCGGCTGCGGCGAGCGGTATCAGCTCACTCAAAGGCGGTAATACGGTTATCCACAGAATCAGGGGATAACGCAGGAAAGAACATGTGAGCAAAAGGCCAGCAAAAGCCCAGGAACCGTAAAAAGGCCGCGTTGCTGGCGTTTTTCCATAGGCTCCGCCCCCCTGACGAGCATCACAAAAATCGACGCTCAAGTCAGAGGTGGCGAAACCCGACAGGACTATAAAGATACCAGGCGTTTCCCCCTGGAAGCTCCCTCGTGCGCTCTCCTGTTCCGACCCTGCCGCTTACCGGATACCTGTCCGCCTTTCTCCCTTCGGGAAGCGTGGCGCTTTCTCATAGCTCACGCTGTAGGTATCTCAGTTCGGTGTAGGTCGTTCGCTCCAAGCTGGGCTGTGTGCACGAACCCCCCGTTCAGCCCGACCGCTGCGCCTTATCCGGTAACTATCGTCTTGAGTCCAACCCGGTAAGACACGACTTATCGCCACTGGCAGCAGCCACTGGTAACAGGATTAGCAGAGCGAGGTATGTAGGCGGTGCTACAGAGTTCTTGAAGTGGTGGCCTAACTACGGCTACACTAGAAGGACAGTATTTGGTATCTGCGCTCTGCTGAAGCCAGTTACCTTCGGAAAAAGAGTTGGTAGCTCTTGATCCGGCAAACAAACCACCGCTGGTAGCGGTGGTTTTTTTGTTTGCAAGCAGCAGATTACGCGCAGAAAAAAAGGATCTCAAGAAGATCCTTTGATCTTTTCTACGGGGTCTGACGCTCAGTGGAACGAAAACTCACGTTAAGGGATTTTGGTCATGAGATTATCAAAAAGGATCTTCACCTAGATCCTTTTAAATTAAAAATGAAGTTTTAAATCAATCTAAAGTATATATGAGTAAACTTGGTCTGACAGTTACCAATGCTTAATCAGTGAGGCACCTATCTCAGCGATCTGTCTATTTCGTTCATCCATAGTTGCCTGACTCCCCGTCGTGTAGATAACTACGATACGGGAGCGCTTACCATCTGGCCCCAGTGCTGCAATGATACCGCGAGACCCACGCTCACCGGCTCCAGATTTATCAGCAATAAACCAGCCAGCCGGAAGGGCCGAGCGCAGAAGTGGTCCTGCAACTTTATCCGCCTCCATCCAGTCTATTAATTGTTGCCGGGAAGCTAGAGTAAGTAGTTCGCCAGTTAATAGTTTGCGCAACGTTGTTGGCATTGCTACAGGCATCGTGGTGTCACTCTCGTCGTTTGGTATGGCTTCATTCAGCTCCGGTTCCCAACGATCAAGGCGAGTTACATGATCCCCCATGTTGTGCAAAAAAGCGGTTAGCTCCTTCGGTCCTCCGATCGTTGTCAGAAGTAAGTTGGCCGCAGTGTTATCACTCATGGTTATGGCAGCACTGCATAATTCTCTTACTGTCATGCCATCCGTAAGATGCTTTTCTGTGACTGGTGAGTACTCAACCAAGTCATTCTGAGAATAGTGTATGCGGCGACCGAGTTGCTCTTGCCCGGCGTCAATACGGGATAATAGTGTATCACATAGCAGAACTTTAAAAGTGCTCATCATTGGAAAACGTTCTTCGGGGCGAAAACTCTCAAGGATCTTACCGCTGTTGAGATCCAGTTCGATGTAACCCACTCGTGCACCCAACTGATCTTCAGCATCTTTTACTTTCACCAGCGTTTCTGGGTGAGCAAAAACAGGAAGGCAAAATGCCGCAAAAAAGGGAATAAGGGCGACACGGAAATGTTGAATACTCATACTCTTCCTTTTTCAATGGGTAATAACTGATATAATTAAATTGAAGCTCTAATTTGTGAGTTTAGTATACATGCATTTACTTATAATACAGTTTTTTAGTTTTGCTGGCCGCATCTTCTCAAATATGCTTCCCAGCCTGCTTTTCTGTAACGTTCACCCTCTACCTTAGCATCCCTTCCCTTTGCAAATAGTCCTCTTCCAACAATAATAATGTCAGATCCTGTAGAGACCACATCATCCACGGTTCTATACTGTTGACCCAATGCGTCTCCCTTGTCATCTAAACCCACACCGGGTGTCATAATCAACCAATCGTAACCTTCATCTCTTCCACCCATGTCTCTTTGAGCAATAAAGCCGATAACAAAATCTTTGTCGCTCTTCGCAATGTCAACAGTACCCTTAGTATATTCTCCAGTAGATAGGGAGCCCTTGCATGACAATTCTGCTAACATCAAAAGGCCTCTAGGTTCCTTTGTTACTTCTTCTGCCGCCTGCTTCAAACCGCTAACAATACCTGGGCCCACCACACCGTGTGCATTCGTAATGTCTGCCCATTCTGCTATTCTGTATACACCCGCAGAGTACTGCAATTTGACTGTATTACCAATGTCAGCAAATTTTCTGTCTTCGAAGAGTAAAAAATTGTACTTGGCGGATAATGCCTTTAGCGGCTTAACTGTGCCCTCCATGGAAAAATCAGTCAAGATATCCACATGTGTTTTTAGTAAACAAATTTTGGGACCTAATGCTTCAACTAACTCCAGTAATTCCTTGGTGGTACGAACATCCAATGAAGCACACAAGTTTGTTTGCTTTTCGTGCATGATATTAAATAGCTTGGCAGCAACAGGACTAGGATGAGTAGCAGCACGTTCCTTATATGTAGCTTTCGACATGATTTATCTTCGTTTCCTGCAGGTTTTTGTTCTGTGCAGTTGGGTTAAGAATACTGGGCAATTTCATGTTTCTTCAACACTACATATGCGTATATATACCAATCTAAGTCTGTGCTCCTTCCTTCGTTCTTCCTTCTGTTCGGAGATTACCGAATCAAAAAAATTTCAAAGAAACCGAAATCAAAAAAAAGAATAAAAAAAAAATGATGAATTGAATTGAAAAGCTAGCTTATCGATGATAAGCTGTCAAAGATGAGAATTAATTCCACGGACTATAGACTATACTAGATACTCCGTCTACTGTACGATACACTTCCGCTCAGGTCCTTGTCCTTTAACGAGGCCTTACCACTCTTTTGTTACTCTATTGATCCAGCTCAGCAAAGGCAGTGTGATCTAAGATTCTATCTTCGCGATGTAGTAAAACTAGCTAGACCGAGAAAGAGACTAGAAATGCAAAAGGCACTTCTACAATGGCTGCCATCATTATTATCCGATGTGACGCTGCAGCTTCTCAATGATATTCGAATACGCTTTGAGGAGATACAGCCTAATATCCGACAAACTGTTTTACAGATTTACGATCGTACTTGTTACCCATCATTGAATTTTGAACATCCGAACCTGGGAGTTTTCCCTGAAACAGATAGTATATTTGAACCTGTATAATAATATATAGTCTAGCGCTTTACGGAAGACAATGTATGTATTTCGGTTCCTGGAGAAACTATTGCATCTATTGCATAGGTAATCTTGCACGTCGCATCCCCGGTTCATTTTCTGCGTTTCCATCTTGCACTTCAATAGCATATCTTTGTTAACGAAGCATCTGTGCTTCATTTTGTAGAACAAAAATGCAACGCGAGAGCGCTAATTTTTCAAACAAAGAATCTGAGCTGCATTTTTACAGAACAGAAATGCAACGCGAAAGCGCTATTTTACCAACGAAGAATCTGTGCTTCATTTTTGTAAAACAAAAATGCAACGCGACGAGAGCGCTAATTTTTCAAACAAAGAATCTGAGCTGCATTTTTACAGAACAGAAATGCAACGCGAGAGCGCTATTTTACCAACAAAGAATCTATACTTCTTTTTTGTTCTACAAAAATGCATCCCGAGAGCGCTATTTTTCTAACAAAGCATCTTAGATTACTTTTTTTCTCCTTTGTGCGCTCTATAATGCAGTCTCTTGATAACTTTTTGCACTGTAGGTCCGTTAAGGTTAGAAGAAGGCTACTTTGGTGTCTATTTTCTCTTCCATAAAAAAAGCCTGACTCCACTTCCCGCGTTTACTGATTACTAGCGAAGCTGCGGGTGCATTTTTTCAAGATAAAGGCATCCCCGATTATATTCTATACCGATGTGGATTGCGCATACTTTGTGAACAGAAAGTGATAGCGTTGATGATTCTTCATTGGTCAGAAAATTATGAACGGTTTCTTCTATTTTGTCTCTATATACTACGTATAGGAAATGTTTACATTTTCGTATTGTTTTCGATTCACTCTATGAATAGTTCTTACTACAATTTTTTTGTCTAAAGAGTAATACTAGAGATAAACATAAAAAATGTAGAGGTCGAGTTTAGATGCAAGTTCAAGGAGCGAAAGGTGGATGGGTAGGTTATATAGGGATATAGCACAGAGATATATAGCAAAGAGATACTTTTGAGCAATGTTTGTGGAAGCGGTATTCGCAATGGGAAGCTCCACCCCGGTTGATAATCAGAAAAGCCCCAAAAACAGGAAGATTGTATAAGCAAATATTTAAATTGTAAACGTTAATATTTTGTTAAAATTCGCGTTAAATTTTTGTTAAATCAGCTCATTTTTTAACGAATAGCCCGAAATCGGCAAAATCCCTTATAAATCAAAAGAATAGACCGAGATAGGGTTGAGTGTTGTTCCAGTTTCCAACAAGAGTCCACTATTAAAGAACGTGGACTCCAACGTCAAAGGGCGAAAAAGGGTCTATCAGGGCGATGGCCCACTACGTGAACCATCACCCTAATCAAGTTTTTTGGGGTCGAGGTGCCGTAAAGCAGTAAATCGGAAGGGTAAACGGATGCCCCCATTTAGAGCTTGACGGGGAAAGCCGGCGAACGTGGCGAGAAAGGAAGGGAAGAAAGCGAAAGGAGCGGGGGCTAGGGCGGTGGGAAGTGTAGGGGTCACGCTGGGCGTAACCACCACACCCGCCGCGCTTAATGGGGCGCTACAGGGCGCGTGGGGATGATCCACTAGT

# Supplementary Figures


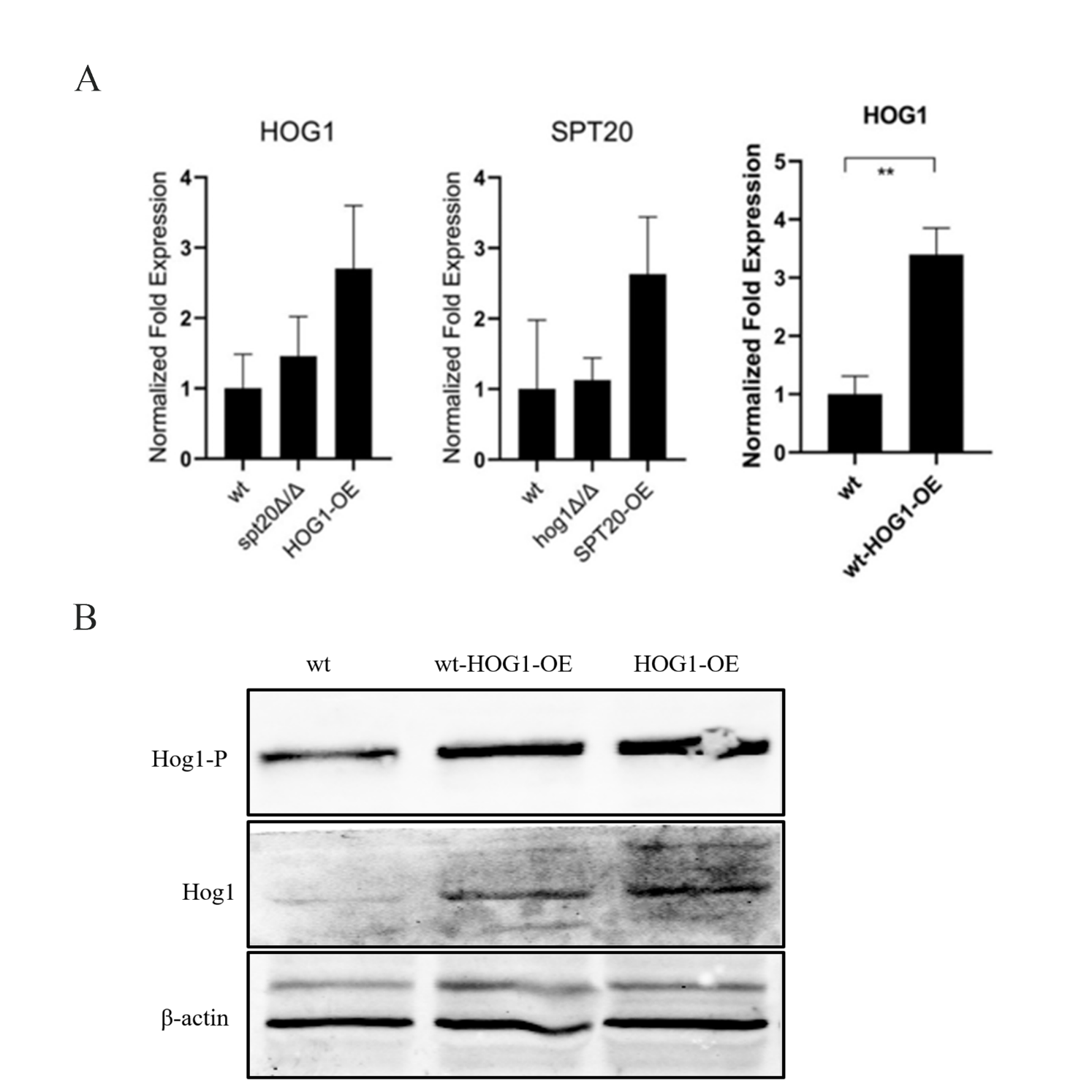


**Supplementary Figure 1. (A)** Quantitative reverse transcription-PCR analysis was used to measure the target gene expression level of HOG1-OE strain in the *spt20Δ/Δ* mutant background, SPT20-OE strain in the *hog1Δ/Δ* mutant background, and wt-HOG1-OE strain in the wild type background, respectively. Expression levels were normalized to *TDH3*. Fold changes between strains were normalized to wild type strain, which was adjusted to a value of 1. The experiment was repeated three times independently. **(B)** Western blotting was used to examine the levels of phosphorylated Hog1 and total Hog1 in the *HOG1* overexpression strains with specific antibodies. The beta actin antibody was used as the loading control. Two independent replicates of the experiment were performed.


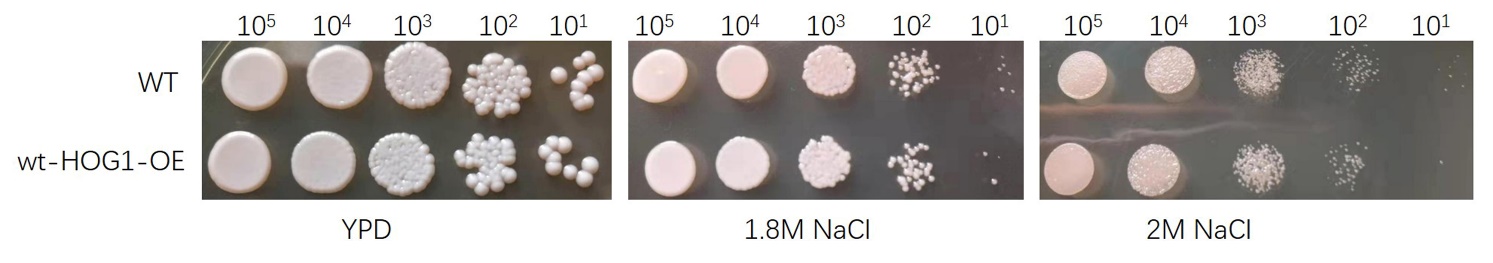


**Supplementary Figure 2.** Ten-fold serial dilutions of cells were spotted on YPD plate or YPD supplemented with NaCl. Cells were grown at 30℃ and then photographed after 2 days. The experiment was repeated on 3 independent occasions.


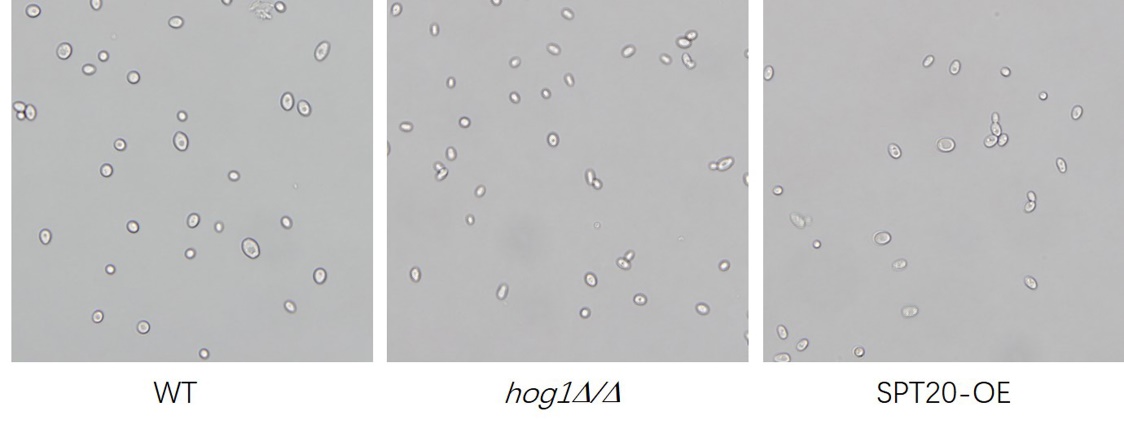


**Supplementary Figure 3.** Wild type, *hog1Δ/Δ*, and SPT20-OE strains were grown overnight in YPD medium, then the morphology of cells was photographed.


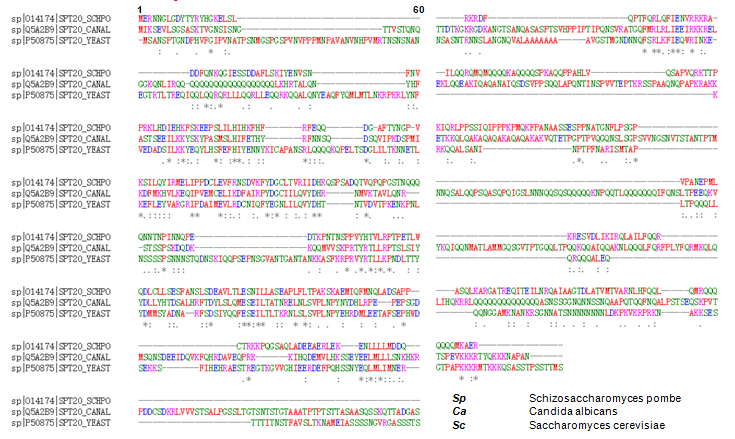


**Supplementary Figure 4.** Amino acid sequence alignment of Spt20 in *C. albicans*, *S. cerevisiae* and *S. pombe*.
